# Urinary and Plasma Antioxidants in Behavioral Symptoms of Individuals With Autism Spectrum Disorder

**DOI:** 10.3389/fpsyt.2021.684445

**Published:** 2021-09-03

**Authors:** George Imataka, Kunio Yui, Yuki Shiko, Yohei Kawasaki, Hitomi Sasaki, Ryoichi Shiroki, Shigemi Yoshihara

**Affiliations:** ^1^Department of Pediatrics, Dokkyo Medical University, Mibu, Japan; ^2^Department of Urology, Fujita Health University, Toyoake, Japan; ^3^Clinical Research Center, Chiba University Hospital, Chiba, Japan

**Keywords:** autism spectrum disorder, urinary total antioxidant capacity, HEL, 8-OHdG, plasma ceruloplasmin, transferrin and superoxide dismutase, aberrant behavior checklist

## Abstract

The balance between antioxidant capacity and oxidative stress-induced free radicals may be crucial in the pathophysiological development factor of autism spectrum disorder (ASD). We measured the following urinary and plasma biomarker levels of oxidative stress and antioxidants. As urinary biomarkers, (1) hexanoyl-lysine (HEL), which is a new biomarker of oxidative stress, (2) the total antioxidant capacity (TAC), and (3) 8-hydroxy-2′-deoxyguanosine (8-OHdG), as a product of oxidative modifications to DNA; and the plasma levels of (4) the antioxidant protein superoxide dismutase (SOD), which is the crucial defense again oxygen reactive species, and (5) transferrin and (6) ceruloplasmin, which are biomarkers of iron and copper neurotransmission and oxidant-antioxidant systems. We examined the relationship between these urinary and plasma biomarkers and behavioral symptoms in 19 individuals with ASD (mean age, 10.8 ± 5.2 years) and 10 age-matched healthy controls (mean age, 14.2 ± 7.0 years). Behavioral symptoms were estimated using the Aberrant Behavior Checklist (ABC). Urinary TAC levels were significantly lower, whereas urinary HEL levels were significantly increased in the ASD group as compared with the control group. The five ABC subscale and total scores were significantly raised in the autism group than in the control group. The results of a linear regression analysis revealed that plasma SOD levels may be a more accurate predictor of differences in ABC scores between individuals with ASD and control individuals. The present study firstly revealed the important findings that the cooperation between the urinary antioxidant TAC and plasma SOD levels may contribute to the ABC subscale scores of stereotypy. Urinary TAC activity and antioxidant protein SOD may be associated with incomplete mineral body store and antioxidant-related transcription factor and browning reactions. Consequently, a critical imbalance between TAC urinary levels and plasma SOD levels may be an important contributor to autistic behavioral symptoms.

## Introduction

A lot of evidence indicated that interactions between genetic and environmental factors at the time of early childhood are critically important in the development of autism spectrum disorders (ASD) (1). A lack of balance between the excessive production of reactive oxygen species and antioxidant capacity may contribute to the pathophysiology of ASD in predisposed individuals (2, 3). Recent ASD studies reported a reduced total antioxidant capacity (TAC) (3), higher levels of 8-hydroxy-2′-deoxyguanosine (8-OHdG), a biomarker of oxidative modifications to DNA (4), and elevated urinary hexanoyl-lysine (HEL) levels (5, 6). Urinary TAC levels are a measure of oxidative stress (7), while plasma superoxide dismutase (SOD) levels are a biomarker of the antioxidant status (8). Although a series of oxidative stress-related biomarkers, including urinary HEL, TAC, and 8-OHdG levels, may provide important and useful information on brain damage induced by oxidative stress (6), they have not yet been examined in detail. Recent studies on ASD reported significantly lower urinary TAC levels, indicating heightened vulnerability to oxidative damage (3) and significantly increased urinary 8-OHdG concentrations (7). Urinary HEL levels were found to be heightened in children with ASD, on the other hand, higher HEL levels associated with the hyperactivity component of the Childhood Autism Rating Scale (5). We already reported significantly lower levels of urinary TAC (9) and significantly higher levels of urinary HEL (10) with no significant differences in 8-OHdG levels in the two groups (8, 9, 11). Nevertheless, the relationships between core ASD behavioral symptoms and urinary HEL, TAC, and 8-OHdG levels have not yet been elucidated.

Plasma (9) and serum (10, 12) levels of copper-binding antioxidant ceruloplasmin (Cp) and plasma (9) or serum (12) levels of iron-binding protein transferrin (Tf) have been identified as pathophysiological factors. Furthermore, erythrocyte (10, 13, 14) and plasma (15, 16) levels of SOD crucially contribute to the pathophysiology of ASD. The relationships of plasma antioxidant proteins, such as Tf, Cp, and SOD, to urinary oxidative stress biomarkers and TAC remain unclear.

We previously reported that increased plasma levels of docosahexaenoic acid (DHA)/arachidonic acid (ARA) and eicosapentaenoic acid (EPA)/ARA may be associated with reduced plasma levels of neuroprotective properties of Cp, diminishing the defensive activity against brain damage, and may thus impart to the pathophysiology of autistic behaviors in individuals with ASD (9, 15). Importantly, this study firstly examined the role of a set of urinary and plasma oxidative stress-related biomarkers, and added antioxidant activity possessed essential polyunsaturated fatty acids. Furthermore, this study explored the association of oxidative stress-related biomarkers between these urinary and plasma biomarkers in ASD.

The enzymes cyclooxygenase (COX), lipoxygenase (LOX), and cytochrome P450 (CYP) have been shown to convert ARA into eicosanoids (16). As the antioxidant effect is related to eicosanoid synthesis regulation (17), decreased plasma ARA levels may reduce the antioxidant capacity.

Regarding the antioxidant capacity of essential fatty acids, DHA plays an important role in brain development and function and has also been shown to enhance the antioxidant and cognitive activities of the brain (18). A previous study demonstrated that DHA altered antioxidant enzyme activity in a valproic acid-induced rat model of ASD (19). A pretreatment with EPA prevented lipid peroxidation by inhibiting lipid oxidation (20) and also enhanced the antioxidant capacity (21). Furthermore, α-linolenic acid (ALA) exhibits antioxidant activity (22). Linolenic acid (LA) is an antioxidant that is present in the human diet (22), and γ-linolenic acid (GLA) has antioxidant potential of bioprocessed substrates (23). Dihomo-γ-linolenic acid (DGLA) has also been shown to exhibit antioxidant activity (24).

Based on these findings, we examined important avenues in research on the role of urinary and plasma antioxidants in the behavioral symptoms of individuals with ASD. The aims of the present study were 3-fold: we examined the associations between the urinary concentrations of HEL, TAC, and 8-OHdG, and plasma levels of Cp, Tf, SOD, and PUFAs, such as ARA, DHA, EPA, LA, ALA, GLA, and DGLA; their associations among the core symptoms of behavioral symptoms in individuals with ASD; and the relationship between urinary TAC levels and plasma SOD levels because SOD 1 is a powerful antioxidant enzyme that binds copper and zinc ions under neurodegenerative conditions (25).

This is a first study of the role of a set of urinary and plasma oxidative stress-related biomarkers and the association of oxidative stress-related biomarkers between these urinary and plasma biomarkers in ASD.

Previous clinical studies reported that urinary TAC levels (26) and the serum antioxidant capacity (26) were affected by a tryptophan-enriched diet and Mediterranean diet, respectively; therefore, we assessed daily food and nutrient intakes by using a semiconstructive questionnaire for the Japanese participants (DHQ) [the junior high school version (DHQ15)].

## Methods

### Participants

Participants comprised 29 young and physically healthy individuals. These participants were recruited to Fujimoto Medical Clinic in Kobe city, Japan and Dokkyo Medical University in Japan between May 2018 and October 2019 *via* a local advertisement. They and their mothers applied to this study to obtain information on their children's urinary antioxidant capacity.

Diagnosis of ASD was conducted using the *Diagnostic and Statistical Manual of Mental Disorders-V* (DSM-V) criteria (27) and certified under the Autism Diagnostic Interview-Revised (ADI-R). These diagnostic procedures were internationally agreed diagnostic classification systems according to ICD 10 Research Diagnostic Guidelines. Diagnostic interviews were conducted with all individuals who were assessed as possible ASD among the 29 participants. Diagnostic procedures were conducted by the psychiatrist (K.Y.) and pediatrician (G.I.) who were professional medical doctors in the field of developmental disorders. Among the 29 participants, 19 were diagnosed with ASD (12 males and seven females, mean age: 10.8 ± 5.2 years old, age range: 6–15 and 22 years old in one subject), and the remaining 10 were normal healthy controls (six males and four females, mean age: 14.2 ± 7.0 years old, age range: 5–17 and 21 years old in one control subjects). The 10 normal controls were estimated to be physically and mentally healthy during initial physical and mental screening tests before the study. The 19 individuals with ASD exhibited the core symptoms of the DSM-V diagnostic criteria for ASD without any abnormal neurological symptoms (e.g., seizures or neurological diseases). The 19 individuals with ASD and 10 healthy control subjects were matched for food intake, age, sex, and full intelligent quotient (IQ) scores (Table 1). Any other abnormalities were detected in physical (resting blood pressure and pulse rate) or clinical laboratory examinations (hematology and plasma chemistry, including plasma fatty acids) in the two groups at initial health screening tests before the study. The IQs of these individuals were assessed by using the Wechsler Intelligence Scale (WISC-IV) for children and adolescents aged 6–16 years old or the respective scale for adults (WAIS-R) (Table 1). Comorbid neuropsychiatric diseases were explored using the Structured Clinical Interview for DSM-IV Axis I Disorders (SCID). Additional criteria of inclusion were as follows: (a) the absence of any other medical or comorbid psychiatric diseases; (b) a baseline verbal or full IQ >70 (28) or the respective scale for adults (WAIS-R) because subjects with high-functioning ASD were estimated to have a total IQ of at least 70 (29); and (c) no treatment with antidepressants, anxiolytic medications, or neuroleptics within 3 months prior to the initiation of the present study.

**Table 1:** Clinical characteristics and urinary and plasma variables, and ABC scores in ASD control groups.

	**ASD** (***N*** **= 19)**	**Controls** (***N*** **= 10)**	***U***	***p*** **-value**
Age (years)	10.8 ± 5.2	14.2 ± 7.0	72.50	0.32
Sex ratio	12/7	3/7		
Full scale IQ	90.0 ± 31.9	117.4 ± 21.1	17.50	0.04^*^
ADI-R scores				
Social interaction	13.2 ± 6.3			
Abnormalities in communication	7.1 ± 5.6			
Restricted, repetitive behavior	11.3 ± 6.2			
Urinary biomarkers				
8-Hydroxy-2′-deoxyguanosine (ng/mgCre)	10.98 ± 5.15	10.45 ± 3.81	92.50	0.91
Hexanoyl-lysine (pmol/mgCre)	77.79 ± 3 1.02	52.99 ± 27.20	50.00	0.04^*^
Total antioxidant capacity (μMUric acid equivalents)	29,983.33 ± 986.03	4,263.04 ± 1,247.96	48.00	0.04^*^
Plasma biomarker				
Ceruloplasmin (mg/dl)	26.29 ± 7.57	27.50 ± 4.88	94.00	0.98
Transferrin (mg/dl)	283.74 ± 36.93	266.40 ± 37.23	74.00	0.35
Superoxide dismutase (mg/dl)	3.93 ± 3.25	4.69 ± 4.13	86.00	0.70
Arachidonic acid (%)	181.08 ± 44.55	174.90 ± 50.49	90.00	0.84
Eicosapentaenoic acid (%)	30.92 ± 24.22	18.56 ± 11.95	58.00	0.09
Docosahexaenoic acid (%)	91.38 ± 43.72	82.07 ± 36.42	81.00	0.54
Linoleic acid (%)	692.50 ± 206.48	761.05 ± 174.89	77.00	0.43
α-Linoleic acid (%)	16.02 ± 6.35	16.60 ± 7.12	94.50	0.98
γ-Linoleic acid (%)	7.51 ± 3.60	7.64 ± 2.09	84.50	0.64
Dihomo-γ-linolenic acid (%)	32.97 ± 9.37	32.33 ± 5.38	89.50	0.80
Aberrant Behavior Checklist scores				
Irritability	14.89 ± 7.08	0.80 ± 1.14	0.00	<0.00
Lethargy	22.05 ± 9.14	0.40 ± 0.97	1.50	<0.00
Stereotypy	5.42 ± 4.68	0.40 ± 0.70	14.00	<0.00
Hyperactivity	22.05 ± 10.39	1.00 ± 2.21	1.00	<0.00
Excess speech	5.11 ± 2.94	0.30 ± 0.67	10.00	<0.00
Total scores	69.11 ± 27.43	2.70 ± 4.72	0.00	<0.00

*ASD, autism spectrum disorder*.

* *P < 0.05*.

The present study was conducted according to the Code of Ethics of the World Medical Association (Declaration of Helsinki) for experiments involving humans. It was performed with the approval of the Ethics Committee of Dokkyo Medical University and the Fujimoto Medical Clinic in Kobe city, Japan. These ethics committees were registered with the Pharmaceuticals and Medical Devices Agency of Japan to register IRB information (http://www.info.pmda.go.jp/). The majority of subjects in this study were under the legal age of 20 years; therefore, we obtained parental permission and assessed data on these individuals (e.g., recognizing whether each participant's urinary TAC and plasma SOD levels were within the standard values according to SRL Inc., Tokyo, Japan). The standard values of TAC, HEL, and 8-OHdG levels were within the standard ranges established by the Department of Pediatrics, Tokyo Metropolitan Fuchu Medical Center for the Disabled, Tokyo, Japan. Written informed consent was given by the participants and/or their parents. This study was registered at the Clinical Trials Registry, Japan Medical Association, June 14, 2010; renewal, December 31, 2015; ID: JMA-IIA00162.

### Management of Food Intake and Assessment of Nutrient Intake

TAC levels in urine may be influenced by food intake (30, 31). Furthermore, plasma fatty acid levels may be confounded by a prior dietary intake (28). To manage dietary intake, all 29 subjects received the “Japanese Food Guide” (32). Moreover, to assess daily food and nutrient intakes, a semiconstructive questionnaire for the Japanese participants (DHQ) was performed using the DHQ15 (DHQ Support Center in Tokyo, Japan (http://www.ebnjapan.org/). DHQ15 consists of 72 questions on the intake frequency of 150 various food and beverage items and cooking methods. The DHQ15 was completed 1 month before the study on randomly selected subsamples of 10 individuals with ASD and 10 normal controls. The estimated intake of nutrients was calculated using a dedicated program for the DHQ system (DHQ Support Center, Tokyo, Japan) (33). The validity of DHQ15 in young adolescents has been already verified (34).

### Measures for Small Sample Size

As the small sample size in the present study, the present findings might not be generalizable. Therefore, efforts are needed for comparing with larger sample size and better controlled studies (35). Furthermore, the mean intraindividual variability in plasma and urine variables is expressed as coefficients of variation which was assessed to the reliability of data (36). Therefore, the coefficients of variation were used to analyze the reliability of plasma and urine analyses.

### Urinary HEL, TAC, and 8-OHdG Levels

Their blood and urine sample using clean voided methods were collected in the order of medical consultation. *Voided* urine was gathered and immediately held at −80°C until analyzed. After the procedures of dissolution, urine samples were centrifuged to remove all insoluble materials. Specialists at the Department of Pediatrics, Tokyo Metropolitan Fuchu Medical Center for the Disabled (Tokyo, Japan) measured urinary HEL, 8-OHdG, and TAC levels. The standard values for TAC, HEL, and 8-OHdG levels were within normal ranges (TAC, 3,700–4,000; HEL, 50–125; 8-OHdG, 9.0–9.7 μM uric acid equivalents in eight normal healthy subjects aged 6–37 years) (37).

### Urinary HEL Levels

Urinary HEL levels were measured in duplicate using a competitive ELISA kit (Japan International Cooperation Agency-JICA, Shizuoka, Japan) (38).

### Urinary 8-OHdG Levels

Void urine were centrifuged, and the supernatants obtained after dilution were used in duplicate for assessments with a competitive enzyme-linked immunosorbent assay kit (8-OHdG check ELISA kit, JalCA, Japan Institute for the Control of Aging, Shizuoka, Japan). Results were then corrected to the urinary concentration of creatinine, and urinary 8-OHdG/creatinine levels were used in subsequent analyses (39).

### Urinary TAC

As described in the instruction manual by the manufacturer (Oxford Biomedical Research), urinary TAC levels were assessed using ELISA (40). This assay measures TAC levels in samples based on the combined activities of the constituents (Oxford Biomedical Research).

### Measurement of Plasma SOD, Cp, and Tf Levels

Whole blood samples were collected by venipuncture into EDTA tubes after a 5-h fast and immediately placed on ice. Although a 9–12-h fast before triglyceride measurements was previously reported to be appropriate (41), another study indicated that fasting for 8 h was sufficient. Besides triglycerides, lipid parameters without fasting are useful when examining for dyslipidemia in children (42) and plasma lipid profiles may be evaluated in non-fasted blood samples (43). Therefore, the 5-h fast employed in the present study was acceptable. After 20 min of supine rest in a quiet room to minimize the effects of circadian variations, blood was collected from participants at 11:00 am−12:30 pm. Samples were kept at −80°C for the later analysis of plasma SOD, Cp, and Tf levels at a clinical analytical laboratory (SRL Inc., Tokyo, Japan).

### Plasma SOD Levels

SOD concentration in plasma were assayed from the rate of decrease in nitrite produced by hydroxylamine and superoxide anions based on the nitrite method using a Versa max instrument (Molecular Devices Co., Tokyo, Japan). Human plasma was assayed using a SOD Assay Kit (Takara Bio, Tokyo, Japan) based on the cytochrome *c* method, and plasma SOD levels were expressed as units per milliliter. The sensitivity of the assay was 0.3 U/ml. Intraassay and interassay coefficients were 2.11 and 2.10 U/ml, respectively.

### Plasma Cp Concentration

A Bering BN II Nephelometer (Siemens Healthcare Diagnostics K.K., USA) was used to estimate plasma Cp levels. Assay sensitivity was 3.0 mg/dl. Intra- and interassay coefficients were 10.2 and 10.1 mg/dl, respectively.

### Plasma Tf Concentration

A standard turbidimetric assay and automated biochemical analyzer (JCA-BM8000 series, JEOL Ltd., Tokyo, Japan) were utilized to estimate plasma Tf levels. Intra- and interassay coefficients were 108.1 and 107.4 mg/dl, respectively.

### Estimation of Autistic Behaviors

The ABC was used to evaluate the behavioral symptoms of the 19 individuals with ASD and 10 normal controls. This scale is a broad-band–estimating instrument that captures a various behavior problems including self-injurious and aggressive behaviors. The ABC was used as a dependent variable in this study to capture a broad range of behavior problems (40). A subscale of ABC is useful for the longitudinal changes of the subject's behavioral problems (44). The ABC can discriminate between disruptive behavior disorders and the behavioral symptoms of ASD (45). The associations between ABC subscales and other behavioral scales, and demographic variables provided useful measures of behavioral problems in ASD (46). The following subscales were employed: (1) irritability (15 items); (2) social withdrawal (16 items); (3) stereotypic behavior (seven items); (4) hyperactivity (16 items); and (5) excessive speech (four items).

### Statistical Analysis

Specialist in statistics was all blinded to the study groups. The assumption of normality was checked. As the data were not normally distributed, the non-parametric Mann–Whitney *U*-test was employed. Multiple regression analyses were performed to investigate potential confounding factors, such as urine and plasma biomarkers, and assessment scales of behavioral symptoms (45). A multiple linear regression was employed to confirm the relationships between the five ABC subscale and total scores, urinary TAC, HL, and 8-OHdG levels, and plasma Cp, Tf, and SOD levels. Statistical analyses were conducted using SPSS version 18.0 (IBM Tokyo, 2009).

## Results

### Study Population

The 19 individuals with ASD showed DSM-V core behavioral and social symptoms: the stereotyped repetitive behaviors (*n* = 11); restricted interests that are abnormal in intensity or focus (*n* = 6); hyperreactivity to sensory input or unusual interests in sensory aspects of the environment (*n* = 2).

The mean ABC total score for our patients was 69.1 ± 27.4 (Table 1). A recent study reported an ABC total score of 23.6 ± 3.7 in 530 children with ASD aged 3–12 years in the autism health network (47). Therefore, our patients exhibited severe autistic behavioral symptoms. No significant differences were observed in age between the two groups (*p* = 0.31).

### CV in Plasma and Urine Variables

The CVs in plasma and urine variables were 41.6% in the ASD group and 37.2% in the control group.

### Urinary Concentrations of Oxidative Stress-Related Biomarkers

Urinary HEL levels were significantly higher (*p* = 0.040), whereas urinary TAC levels were significantly lower (*p* = 0.031) in the 19 individuals with ASD than in the 10 normal controls. No significant differences were observed in urinary 8-OHdG levels or plasma SOD levels between the groups (Table 1).

### Predictor Variables

As shown in Table 2, plasma SOD levels (*B* = 4.160 ± 1.680, β = 0.376; *p* = 0.027) significantly contributed to ABC total scores. Urinary TAC levels (*B* = 0.002 ± 0.001, β = 0.561; *p* = 0.020) and plasma SOD levels (*B* = 0.856 ± 0.286, β = 3.001; *p* = 0.010) contributed to the stereotypy subscale score.

**Table 2:** Results of libear regressuin analysis.

**Model**	**Model**		**Model B**	**Coefficients**	
	***R*** ^**2**^	***p-*** **value**		**Beta**	***p-*** **value**
ABC irritability	0.820	0.004^*^			
Eicosapentaenoic acid			0.216 ± 0.123	0.521	0.099
ABC lethargy	0.850	0.001^*^			
Superoxide dismutase			1.377 ± 0.674	0.380	0.060
ABC stereotypy	0.781	0.021^*^			
Total antioxidant capacity			0.002 ± 0.001	0.516	0.002^*^
Superoxide dismutase			0.858 ± 0.286	0.674	0.010^*^
Dihomo-γ-linolenic acid			0.278 ± 0.144	0.502	0.074
ABC hyperactivity	0.742	<0.000			
Superoxide dismutase			1.355 ± 0.646	0.362	0.055
α-Linoleic acid			0.557 ± 0.303	0.274	0.087
ABC excess speech	0.842	0.002^*^			
Ceruloplasmin			−0.139 ± 0.062	−0.280	0.043^*^
ABC total	0.900	<0.000			
Superoxide dismutase			4.160 ± 1.680	0.376	0.027^*^

*ABC, Aberrant Behavior Checklist*.

* *P < 0.05 significant contribution*.

Collectively, these results suggest that plasma SOD levels and urinary TAC levels are accurate predictors of differences in the biomarkers in the ABC total scores and stereotypy subscale scores, respectively, from the control group (Table 2).

### Assessment of Nutrient Intake

There were no significant differences in weight, height, energy intake, or the intakes of protein (*p* = 1.00), cholesterol (*p* = 0.49), omega-6 (*p* = 0.44), iron *p* = 0.55), copper (*p* = 0.55), omega-3 (*p* = 0.50), or omega-6 PUFAs (*p* = 0.44) between the two groups (Table 3).

**Table 3:** Dietary TAP in the random subsamples that include 10 individuals with 10 individuals with ASD and 10 normal controls.

	**ASD** (***N*** **= 10)**	**Control** (***N*** **= 10)**	***U***	***p***
FRAP				
Non-alcohol beverages				
Green tea	7.13 ± 5.70	8.72 ± 4.17	42.5	0.58
Fruit juice	2.79 ± 4.28	1.89 ± 4.19	31.5	0.17
Vegetables				
Cabbage	0.26 ± 0.26	0.16 ± 0.13	39.5	0.44
Radishes	0.45 ± 0.25	0.31 ± 0.26	33.5	0.22
Tomatoes	0.08 ± 0.08	0.10 ± 0.09	43.0	0.63
Lettuce	0.09 ± 0.05	0.07 ± 0.06	41.0	0.53
Lotus root	1.10 ± 0.77	0.80 ± 0.44	44.5	0.68
Other salted pickles	0.002 ± 0.006	0.17 ± 0.10	8.0	0.21
Sea vegetables	0.57 ± 0.49	0.07 ± 0.07	27.5	0.09
Fruits				
Oranges	0.65 ± 0.78	0.42 ± 0.51	39.5	0.44
Strawberries	1.10 ± 1.78	0.44 ± 048	43.0	0.63
Confectionery				
Chocolate	0.94 ± 0.80	0.29 ± 0.33	20.0	0.02^*^
Japanese sweets with a sweet filling	0.02 ± 0.03	0.04 ± 0.05	49.0	0.97
Jam and Marmalade	0.15 ± 0.13	0.00 ± 0.00	45.0	0.01^*^
Cookies and biscuits	0.06 ± 0.05	0.02 ± 0.02	22.0	0.04^*^
pancake	0.03 ± 0.03	0.02 ± 0.02	41.0	0.53
Japanese noodles	0.39 ± 0.30	0.24 ± 0.23	33.0	0.22
ORAC				
Breakfast cereals	0.63 ± 0.46	0.42 ± 0.35	40.5	0.48
Egg	0.03 ± 0.01	0.02 ± 0.01	35.0	0.28
Meat and meat products	0.17 ± 0.09	0.20 ± 0.20	43.5	0.63
Poultry and poultry products	0.06 ± 0.05	0.07 ± 0.07	42.5	0.58

*The values are mean ± SD*.

*FRAP, ferric-reducing ability of plasma; ORAC, oxygen radical absorbance capacity*.

* *p < 0.05 compared with the controls*.

### Main Findings

Urinary antioxidant biomarker HEL were increased, while, urinary antioxidant biomarker TAP levels were reduced in the ASD group. Consequently, a critical imbalance between urinary TAC levels and plasma SOD levels may be an important contributor to autistic behavioral symptoms.

## Discussion

We calculated the coefficient of variation (CV) (mean/SD) in plasma and urine variables to analyze the reliability (36) of plasma and urine analyses. The CVs in the ASD and control groups were 41.65 and 37.2%, respectively. The JAMA clinical trial report indicated that the CV in plasma variable concentration was 46.1 and 32% in two groups with sunscreen spray and lotion 4.3 and 1.8 ng/ml, respectively (48). Furthermore, a previous study of microRNA expression levels indicated the CV of 20–40% (49). Although the variations in plasma and urine variables were large, the CVs in this study appeared to be reasonable.

In this study, age difference between ASD subjects and controls was 4.5 years (Table 1). These ages in the two groups were not significantly different (*p* = 0.328). A lot of previous studies on randomized clinical trials included subjects with age range of 5–17 years (50) or 18–35 years (51). In a recent clinical report, 1,260 adolescent girls with ages 10–17 years were included in food survey study (52). Thus, age ranges in this study appeared to be reasonable.

This study revealed that TAC concentrations in urine were significantly reduced, while HEL concentrations in urine were significantly increased in the 19 individuals with ASD than in the 10 healthy controls. Furthermore, there were no significant differences in 8-OHdG concentrations in urine between the two groups. The results of a linear regression analysis revealed that plasma SOD and Tf concentrations and urinary TAC concentrations were accurate predictors of differences in the biomarkers and the ABC total scores and subscale scores of stereotypy and excess speech between the ASD and control groups. These results indicate that an important relationship exists between urinary TAC levels and plasma SOD levels, and also that plasma SOD levels contribute to ABC scores (Table 2). Furthermore, plasma SOD levels may be a more accurate predictor of differences in ABC scores between the two groups.

Regarding the role of SOD in ASD, reduced serum SOD levels have been contributed to the pathophysiology and progression of ASD in 96 children, suggesting that SOD is a risk factor for the development of ASD (53). SOD levels were previously shown to be higher in autistic children than in typically developed children, and this increase was associated with the upregularized expression of nitrotyrosine, a marker of oxidative damage, in the immune cells of ASD subjects (54). SOD levels in serum were significantly reduced in 30 children with ASD as compared with those in the control individuals with no severe ASD symptoms, suggesting the utility of reduced serum SOD levels as an early diagnostic biomarker (55). Previous animal studies reported that an injection of SOD induced ASD-like repetitive behavior (56), and that SOD 1 knockout mice exhibited impaired motivational behavior (57). SOD levels were reduced in a rat model of maternal diabetes (58), and SOD 1 genetic mice exhibited bizarre behavior (59). Therefore, SOD may regulate social behavior in animals.

The present results indicated a relationship between plasma SOD levels and autistic behavior, and, thus, provide insights into the contribution of SOD to autistic behavior, such as stereotypy. It is important to note that urinary TAC levels and plasma SOD concentrations were secure indicator to distinguish the ASD group from the control group. These results indicate additional and useful information on the crucial imbalance between heightened oxidative stress and deficit urinary antioxidant systems in the pathophysiological factors in ASD subjects.

Regarding the relationship between urinary TAC levels and plasma SOD levels, accumulating evidence indicates that the imbalance between oxidative stress-induced reactive oxygen species and urinary TAC levels correlates with ASD. A previous report demonstrated that SOD levels were higher in autistic children than in typically developed children, and this increase was associated with the up-regularized expression of nitrotyrosine, a marker of oxidative damage, in the immune cells of ASD subjects (60). Of reference, plasma SOD may affect urinary TAC activity (10). Other clinical reports indicated significant decreases in plasma SOD and urinary TAC concentrations in 47 patients with sarcoma (51). Collectively, these findings revealed concomitant changes in plasma and urine SOD and TAC levels, which is consistent with the present results.

In the present study, the linear regression analysis showed that urinary TAC levels significantly contributed to the ABC subscale scores of stereotypy. Urinary antioxidant levels were previously shown to be significantly lower in autistic children and positively correlated with symptom severity (61). We previously reported reduced urinary TAC levels (9, 11) and increased urinary oxidative stress biomarker HEL (15) in 20 autistic children. Furthermore, significant reductions were observed in urinary TAC levels with increases in the severity of ASD (61). In comparisons with 24 age-matched healthy controls, reduced urinary TAC levels with no consequent heightened urinary catalase activity and total thiol molecules, which are the measures of the antioxidant activities (3), were reported in 29 subjects with ASD with age of 6–12 years (3). Moreover, plasma and erythrocyte TAC levels were significantly lower in 34 adolescent individuals with Asperger syndrome (mean age: 12.89 ± 2.58 years) than in 34 age-matched controls (62). These findings suggest that urinary TAC levels in association with a low detoxifying capacity, as indicated by reduced plasma SOD levels, in ASD individuals contribute to the characteristic behavioral symptoms of ASD. Importantly, this study demonstrated for the first time a relationship between urinary TAC levels and plasma SOD levels that contribute to the characteristic symptoms of ASD, such as stereotypy.

Assessment of daily nutrient intake using DHQ15 revealed no significant differences in the intake of fat, protein, vitamin B2, vitamin B6, vitamin B12, vitamin C, omega-6, or omega-3 PUFAs between randomly selected subsamples of 10 individuals with ASD and those of 10 controls. Regarding the association between dietary intake and urinary TAC levels, virgin olive oil (63) and tryptophan-enriched cereal (26) were increase urinary TAC levels, whereas refined potato starch reduced urinary TAC concentrations (64). Thus, the effects of food intake on urinary TAC levels may vary, and differences between the groups examined did not appear to influence urinary TAC levels. The intake of TAC from chocolate, biscuits, cookies, jam, and marmalade was significantly increased in the ASD group than in the control group. Products of cocoa, such as chocolate, are good sources of dietary antioxidants (65). Cookies containing chocolate chips increased antioxidant capacity (63), Furthermore, biscuits containing 5% cocoa held antioxidant properties (66). Jam and marmalade productions increased the antioxidant capacity (67), and bilberry jams contained antioxidant compounds (68, 69). Although chocolate, cookies, biscuits, jam, and marmalade contained potent antioxidant abilities, urinary TAC levels were significantly lower in the ASD group than in the control group.

Previous studies on the antioxidant capacity in subjects with ASD implied vulnerability to oxidative stress because of an unbalance in intracellular and extracellular antioxidant activities or a chronically reduced detoxifying capacity (62, 70).

A recent study on antioxidant networks indicated that antioxidant enzymes, such as SOD, glutathione peroxidase, and oxidized glutathione, function as an antioxidant network within specific intracellular or extracellular components of the antioxidant system (71). Such vulnerable antioxidant systems may be due to genetic and dietary factor (72) or the integrative concept of balance between oxygen- and capacity-limited thermal tolerances (73). Dysregulation of antioxidant enzymes such as SOD, glutathione peroxidase, and glutathione reductase were included in neutrophils and monocytes as in antioxidant network in peripheral innate immune cells (54).

SOD and catalase were also antioxidant defense system (74), and SOD/catalase activities may contribute to ASD pathophysiology (75). Interestingly, an inverse relationship between SOD activity and catalase due to changes in the toxic effects of polyvinyl chloride has been reported (76). Additionally, reactive oxygen species competes between oxidative stress (2, 77) and contribute to the development of ASD (75). These antioxidant enzymes such as SOD, glutathione peroxidase, glutathione reductase, catalase, and reactive oxygen species (ROS) have been found to contribute to the pathophysiology of ASD (2).

Glutathione depletion and the activities of antioxidant enzymes such as SOD and ROS levels were closely associated with the cytoprotective effects of gamma-aminobutyric acid (GABA) against oxidative stress *via* the increased expression of interleukin 8 related to the transcription factor [nuclear factor erythroid 2-related factor 2 (Nrf2)] (78), which has an antioxidant activity (79), and upregulating expression of phase II antioxidant genes (80). Moreover, formula-induced Maillard reaction was related to SOD, catalase, glutathione peroxidase, and messenger RNA levels of miRNA-21 and miRNA-155 (81). Importantly, accumulating evidence indicated that browning or Maillard reaction, which was induced by oxidative reactions of food and cookie, produce strong activity of antioxidant (82). This reaction may contribute to increased SOD activity (83, 84). Several previous reports on the antioxidant activities in individuals with ASD indicated vulnerability to oxidative stress because of an imbalance in intracellular and extracellular antioxidant capacities (85) or an endogenous reduced detoxifying capacity (62, 70). Such antioxidant defense systems may be involved in the endogenous antioxidant system. A present finding on antioxidant networks indicated that antioxidant enzymes, such as SOD, glutathione peroxidase, and oxidized glutathione may operate as an antioxidant network in the specific intracellular or the extracellular antioxidant system (71).

In the present study, the linear regression analysis indicated that plasma Cp levels contributed to the ABC subscale scores of Excess speech. Plasma Cp levels were previously shown to be reduced in 28 autistic children with a mean age of 13.2 years (12). Reduced plasma Cp levels may induce or be a consequence of neurodegeneration, such as Alzheimer's disease, while decreased plasma SOD activity may exacerbate defective Cp activity, indicating an intrinsic relationship between plasma Cp and SOD levels (86). Elevated serum Cp levels have been associated with impulsiveness in patients with Parkinson's disease (87). Cerebrospinal fluid Cp can be induced in neurodegenerative disorders (88). Collectively, these findings implicate Cp in neurodegeneration in ASD.

The present study had some limitations: (a) plasma and urinary concentrations of glutathione peroxidation (GPX) are frequently used as antioxidant in clinical studies (89, 90). However, urinary GPX concentrations were sensitive to selenium (79, 91). Thus, urinary GPX may not reflect serum total antioxidant capacity. Additionally, the present study revealed an informative set of antioxidant biomarkers including HEL and 8-OHdG concentrations in urine, and Cp, Tf, and SOD concentrations in plasma, for the first time discovered important findings on impaired antioxidant systems; (b) the male/female ratio in the present study was 13/7 (1.8/1.0), which is consistent with several clinical studies reported previously (92). Furthermore, the ratio of normal controls to cases in the present study was (2:1), which was similar to that in a randomized clinical trial on a new drug (experimental group/control group ratio of 2:1) (93). (c) Although there is no significant difference in ages between ASD and control groups, the age range of both groups are different. Of reference, a lot of previous clinical studies indicated large age ranges in patients and healthy controls. Several clinical studies, for example, showed that 34.68 ± 15.43 years old in 65 patients with diabetes mellitus and 47.33 ± 19.58 years old in 27 healthy controls (94), and that 70.5 ± 2.1 years old in 50 heart failure patients in New York, and 47.9 ± 9.7 years old in 28 healthy university population (95); (d) given the small sample sizes, this study employed two types of measures: first, an endogenous antioxidant system is needed for comparing larger sample size and better-controlled studies described in the Journal of American Medical Association (JAMA) (96). Of reference, the present findings were supported by previous studies (10, 12–15, 97, 98). The present findings on increased urinary HEL levels and decreased urinary TAC levels (8, 11) were supported by previous studies. According to the previous studies, the CV in this study is reasonable (49, 50). To measure small sample size in this study, the mean intraindividual variability in plasma and urine variables is expressed as CV (36).

## Conclusion

The present findings are the first to report the reduced urinary TAC levels and increased urinary HEL levels in individuals with ASD without significant changes in urinary 8-OHdG levels. Importantly, the cooperation between the urinary antioxidant TAC and plasma SOD levels may contribute to stereotypy. Therefore, the role of redox regulation in both plasma SOD and urinary TAC may play an important role in autistic stereotypy. Additionally, the results of linear regression analysis revealed that SOD concentrations in plasma are a more adequate parameter for discriminating the ASD group from the control group. Therefore, the generation of ROS and TAC balance was defective in individuals with ASD. Urinary TAC demonstrated that elements and incomplete mineral body store or nitric oxide may be related to urinary activities of urinary TAC. The activities of antioxidant protein SOD were related to transcription factor and browning or Maillard reactions. These present results suggest that the endogenous antioxidant defense system is defective in youth in subjects with ASD.

## Data Availability Statement

The datasets presented in this study can be found in online repositories. The names of the repository/repositories and accession number(s) can be found in the article/supplementary material.

## Ethics Statement

The studies involving human participants were reviewed and approved by the Ethical Committee of Dokkyo Medical University. Written informed consent to participate in this study was provided by the participants' legal guardian/next of kin. The animal study was reviewed and approved by the Ethical Committee of Dokkyo Medical University. Written informed consent was obtained from the owners for the participation of their animals in this study. Written informed consent was obtained from the individual(s) and minor(s)' legal guardian/next of kin, for the publication of any potentially identifiable images or data included in this article.

## Author Contributions

KY and GI conceptualized and designed this study and contributed to the writing of the manuscript. KY, GI, and HS contributed to the collection of the plasma and urinary samples. YK and YS conducted statistical analysis. KY analyzed the data. KY, GI, HS, and RS contributed to the interpretation of the data and results. All of the authors approved the final manuscript and submission to the Frontiers in Psychiatry.

## Conflict of Interest

The authors declare that the research was conducted in the absence of any commercial or financial relationships that could be construed as a potential conflict of interest.

## Publisher's Note

All claims expressed in this article are solely those of the authors and do not necessarily represent those of their affiliated organizations, or those of the publisher, the editors and the reviewers. Any product that may be evaluated in this article, or claim that may be made by its manufacturer, is not guaranteed or endorsed by the publisher.
